# Breastfeeding self-efficacy in terms of sleep quality, perceived social support, depression and certain variables: a cross-sectional study of postpartum women in Turkey

**DOI:** 10.1186/s12884-024-06456-5

**Published:** 2024-04-02

**Authors:** Dilek Konukbay, Emine Öksüz, Gulten Guvenc

**Affiliations:** 1 Department of Pediatric Nursing, University of Health Sciences Turkey, Gulhane Faculty of Nursing, Ankara, Turkey; 2 Department of Psychiatric and Mental Health Nursing, University of Health Sciences Turkey, Gulhane Faculty of Nursing, Ankara, Turkey; 3 Department of Obstetrics and Gynecology Nursing, University of Health Sciences Turkey, Gulhane Faculty of Nursing, Ankara, Turkey; 4 Sağlık Bilimleri Üniversitesi, Gülhane Gülhane Hemşirelik Fakültesi General Tevfik Sağlam Cad, Etlik, Ankara 06018 Türkiye

**Keywords:** Breastfeeding self-efficacy, Postpartum women, Sleep quality, Social support, Depression

## Abstract

**Background:**

Breastfeeding self-efficacy is one of the key factors that affect a healthy and successful breastfeeding process. A mother’s belief regarding her ability to breastfeed is influenced by social and psychological factors. This study aimed to investigate the breastfeeding self-efficacy levels of postpartum women, the factors affecting this, and its relationship with sleep quality, social support and depression.

**Methods:**

This descriptive cross-sectional study was conducted in the pediatric department of a tertiary hospital in Ankara, Turkey. Data were collected from 200 postpartum women using the Breastfeeding Self-Efficacy Scale-Short Form (BSES-SF), the Pittsburgh Sleep Quality Index (PSQI), the Multidimensional Scale of Perceived Social Support (MSPSS) and the Edinburgh Postnatal Depression Scale (EPDS).

**Results:**

The mean scores of the BSES-SF, PSQI, MSPSS and EPDS were 59.05 ± 8.28, 9.18 ± 3.67, 57.82 ± 18.81, and 8.98 ± 5.89, respectively. A statistically significant negative correlation was found among the BSES-SF, EPDS (*r* = -0.445, *p* = 0.001) and PSQI (*r* = -0.612, *p* = 0.004), while a positive correlation was found among the BSES-SF, total MSPSS (*r* **=** 0.341, *p* = 0.036), and family support (*r* **=** 0.373, *p* = 0.014) (*p* < 0.05). In addition, a statistically significant difference was found between the number of births and breastfeeding self-efficacy (F = 3.68; *p* = 0.001). The linear regression analysis revealed that sleep quality (β = -0.491, *p* = 0.001), perceived social support (β = 0.146, *p* = 0.015), family support (β = 0.153, *p* = 0.013), and depression (β = -0.228, *p* = 0.001) emerged as the predictors of breastfeeding self-efficacy.

**Conclusions:**

In this study, the increase in sleep quality and perceived social support positively affected the breastfeeding self-efficacy of postpartum women, while giving birth for the first time and an increase in the risk of depression were negatively affected.

## Background

Breast milk is an ideal food that can meet all the nutritional elements of a baby for the first six months. The World Health Organization (WHO) strongly recommends that infants should solely be breastfed for the first six months [1]. Breastfeeding has many advantages for both mother and baby, including psychological effects [2]. To promote, encourage and support breastfeeding, comprehensive programmes have been carried out in Turkey with the cooperation of the United Nations Children’s Fund (UNICEF) and WHO since 1991. Despite extensive studies, breastfeeding rates are still below the targeted levels both in Turkey and in the world [3, 4]. It is estimated that only 44.0% of infants under six months worldwide have been breastfed [1]. According to the Demographic and Health Survey of Turkey’s 2018 data, 97.0% of babies breastfeed for a certain period, but the ratio of only breastfeeding in the first six months is 41.0% [5]. There is a rich body of literature on the factors that influence breastfeeding, including social and demographic features, mental and physical status, self-efficacy and social support [6–8].

Breastfeeding self-efficacy of the mother plays an important role in maintaining a healthy and successful breastfeeding process [9, 10]. Breastfeeding self-efficacy shows the mother’s thoughts/perception about breastfeeding, whether she will breastfeed, how much effort she will put into it and her possible reactions towards difficulties that may be encountered in the process [11, 12]. It has been stated in the literature that women’s self- efficacy in breastfeeding is influenced by many factors, such as the mother’s previous experiences, the examples she sees in her environment, stress, sleep quality, attitudes towards breast-feeding, knowledge of breast-feeding and physical or mental condition, and her perception of being socially supported [8, 13].

The postpartum period is a significant phase in terms of early initiation and continuation of breastfeeding. It has been reported that one of the major problems confronted by women in this period is insufficient sleep due to interruptions during the night to breastfeed [14]. Inadequate sleep during this period may cause fatigue and adverse effects on breastfeeding self-efficacy [9, 15, 16]. In the literature, it has been reported that breastfeeding self-efficiency is negatively influenced by the quality of sleep of mothers during the postpartum phase [9, 15, 17].

Being socially supported is another significant factor in the mother’s adaptation to the postpartum period. Social support can be described in terms of any kind of moral and material support provided by the inner circle of the individual. Social support positively affects the mother’s mental health and, therefore, the baby’s health by increasing the mother’s sense of competence in the mothering role [7]. Studies have shown that mothers with more social support are more likely to be successful in breastfeeding [7, 8, 18]. Social support can improve self-care and self-confidence, and it may have a positive effect on a person’s physical, psychological and social conditions, which can lead to improved breastfeeding self-efficacy [10, 18]. Depending on hormonal fluctuations, physiological and related processes that occur in the postpartum period affect each woman differently [15, 19, 20]. Studies show that depressive symptoms in the mother in the early postpartum period has a negative impact on the frequency and intervals of breastfeeding. Maternal confidence in breastfeeding is often influenced by depressive symptoms [7, 8].

Other studies have focused on the relationship between variables in the postpartum period, such as type of birth, time to start breastfeeding, fatigue, maternal attachment and sociodemographic properties [13, 21, 22]. However, studies that delve into the correlation of breastfeeding self-efficacy regarding sleep quality, perceived social support and depression in postpartum women are limited. Additionally, research has examined the effects of sleep quality, perceived social support and depression as a single variable or two variables in regard to breastfeeding self-efficacy [7–9, 18]. Among the studies conducted in Turkey, none were found that examined breastfeeding self-efficacy in a multidimensional manner. In this context, this study aimed to examine the correlation between breastfeeding self-efficacy and sleep quality, perceived social support and depression in women in the postpartum period and the influencing factors. Nurses have a significant role in determining the factors that affect the mother’s self-efficacy and controlling it with effective interventions [9]. The results of this study will guide nurses in planning initiatives to boost breastfeeding self-efficacy.

## Methods

### Study design and participants

This was a descriptive and cross-sectional study conducted in the healthy infant unit of the pediatrics department of a tertiary hospital in Ankara, Turkey. In the healthy infant unit, the development of infants between 0 and 12 months is followed. The purposive sampling method was used for sample selection. In order to increase data quality and reliability during the data collection process, care was taken to ensure that the data collection tools were created in accordance with the purpose of the research, the questions were understandable and consistent, and there were no errors in the data. All forms included in the data analysis were fully completed by the participants.

This study included 200 women who brought their babies to the healthy infant unit for follow-up between January and March 2019 and were in the 0–4 months postpartum period. The sample size was determined using the following equation: *n* = *P* × (100 − *P*) × *z*^2^_/_*d*^*2*^ [23]. *P* is the expected prevalence, *d* is the desired sensitivity, and *z* is the appropriate value in the normal distribution. A sample size of 189 was considered representative of this sampling with a level of 95% confidence, 10.0% precision and 50% anticipated prevalence (as there were no accurate reports for breastfeeding rate in postpartum women, and 50.0% is expected to ensure maximum sample size). When the study was conducted, 232 women that brought their babies to the healthy infant unit, although 32 women did not want to participate in the study. The total data of 200 women were analysed (response rate 86.2%). Women who had any difficulties, disabilities or disorders that could restrain breastfeeding, such as risky pregnancies, multiple births, serious illnesses, known birth deformities or those who chose not to nurse their babies, were excluded from the study.

### Data collection tools

Data were collected through the descriptive information form, the Breastfeeding Self-Efficacy Scale-Short Form (BSES-SF), the Pittsburgh Sleep Quality Index (PSQI), the Multidimensional Scale of Perceived Social Support (MSPSS) and the Edinburgh Postpartum Depression Scale (EPDS).

The descriptive information form of the women was arranged by the researchers consistent with the literature. In this form, there were questions to determine the sociodemographic characteristics, social support, sleep and breastfeeding status of women.

Breastfeeding Self-Efficacy Scale-Short Form (BSES-SF) was developed by Dennis (2003) to assess breastfeeding self-efficacy [11]. The lowest score that can be obtained from the scale, which consists of 14 items in total, is 14, and the highest score is 70. A higher score means higher breastfeeding self-efficacy. The Turkish validity and reliability study of the short version of the scale were performed by Alus et al. (2010) [24]. The Cronbach’s α value of the scale was found to be 0.86. The Cronbach’s α value of the scale in this study was found to be 0.88.

Pittsburgh Sleep Quality Index (PSQI) was developed by Buysse et al., (1989) [25]. The Turkish validity and reliability of the index was done by Agargün et al. (1996) [26]. The index has 7 components: Habitual sleep efficiency, sleep time, sleep delay, subjective sleep quality, sleep disturbance, use of sleeping drugs and daytime functions. Each item was graded on a scale from 0 to 3. The sum of the seven component scores ranged from 0 to 21, and the higher the score, the worse the sleep quality. Sleep quality is considered ‘good’ if the total score is ≤ 5, and ‘bad’ if it is > 5. Agargün et al. (1996) reported the Cronbach’s α a value of the scale as 0.80 [26]. The Cronbach’s α value of the scale was found to be 0.73 in this study.

Zimet et al. (1988) developed Multidimensional Scale of Perceived Social Support (MSPSS). Turkish validity and reliability studies were performed by Eker and Arkar (1995) [27, 28]. The scale, which consists of 12 items in total, has three subscales: family, friend, and significant others. The lowest score that can be obtained from the subscales is 4, and the highest score is 28. The lowest score to be obtained from the whole scale is 12, and the highest score is 84.

A high score indicates high perceived social support. Eker and Akar (1995) found the Cronbach’s α value for the total of the scale to be 0.89 [28]. In this study, the Cronbach’s α value of the scale was found to be 0.93.

Edinburgh Postpartum Depression Scale (EPDS) was developed by Cox et al.(1987) to screen postpartum depression in women [29]. In Turkey, validity and reliability studies were done by Engindeniz et al. (1996) and Aydın et al. (2004) [29–31]. From the 10-item scale, the lowest score of 0, and the highest score of 30 can be taken. The cut-off point of the scale is 13. A score of 13 and above on the scale is regarded as a risk group for postnatal depression [30, 31]. Aydın et al. (2004) found the Cronbach’s α value of EPDS to be 0.72 [31]. The Cronbach’s α value of the scale in this study was found to be 0.80.

### Data collection

Data collection forms were administered to each woman by the researchers through a one-on-one interview in the waiting room in the healthy baby department after the examination of the baby and answered by the participants. Each woman was informed about the study’s aim. Completing the forms took approximately 15–20 min.

### Data analysis

Data were analyzed with the Statistical Package for the Social Sciences (SSPS) 21.0 (SPSS Inc., Chicago, IL, USA, 2012). Number, mean, standard deviation and percentage values were used to define the data. In order to establish the nature of the data as parametric or nonparametric, analysis of conformity to normal distribution was performed, and the variables were found to be parametric. Descriptive characteristics were compared with breastfeeding self-efficacy using the independent t-test and the one-way ANOVA test. The Pearson correlation test was used to calculate the correlation between variables, Multiple linear regression analysis was used to identify the influence of sleep quality, perceived social support, and depression on breastfeeding self-efficacy, and *p* < 0.05 was accepted as statistically significant.

## Results

### Descriptive characteristics

The descriptive features of the women who participated in the study are provided in Table 1. The mean age was 29.52 ± 7.96, 52.0% were university graduates, while 59.5% were housewives. After giving birth, 68.0% of the participants had a helper and 57.5% had their mother or sister who stayed with them as an aide. A total of 80.0% of women had a planned pregnancy, 53.0% had vaginal delivery, and 49.5% experienced their first birth. Of the participants, 79.0% indicated that they did not have any problems during pregnancy, and 60.0% experienced no difficulties during breastfeeding. A total of 47.5% of the mothers stated that they received training on breastfeeding, 13.0% of whom received this training from a nurse. In addition, 48.0% stated that they received assistance in breastfeeding and baby care with 58.3% indicating that they received this help from their mothers. It was found that 16.5% of the women in the study had a high risk of postpartum depression, and 16.0% had poor sleep quality (Table 1).

**Table 1:** Descriptive characteristics of the participants (*N* = 200)

Age (Mean ± SD)	29.52 ± 7.96	
	**n**	**%**
**Educational status**		
Primary school	38	19.0
High school	58	29.0
University	104	52.0
**Spouse’s educational status**		
Primaryschool	35	17.5
High School	61	30.5
University	104	52.0
**Current working status**		
Legal postpartum permission	49	24.5
Free postpartum permission	20	10.0
Not working	119	59.5
Leaving work due to giving birth	12	6.0
**Spouse’s employment status**		
Employed	196	98.0
Unemployed	4	2.0
**Status of staying with a relative after birth**		
Yes	136	68.0
I am still staying with my close.	42	21.0
No	22	11.0
**Staying person**		
Mother or sister	115	57.5
Mother-in-law	67	33.5
Other	14	7.0
**Was the pregnancy planned?**		
Planned pregnancy	160	80.0
Unplanned pregnancy	40	20.0
**Mode of delivery**		
Vaginal Birth	106	53.0
C-section	94	47.0
**Number of births**		
I have never given birth before; it is my first birth	99	49.5
It is my second birth	65	32.5
It is my third birth	36	18.0
**Having problems during pregnancy**		
Yes	42	21.0
No	158	79.0
**Having problems with breastfeeding**		
Yes	20	40.0
No	180	60.0
	**n**	**%**
**The person who gives the most help in breastfeeding and baby care**		
Mother	56	58.3
Mother-in-law	19	19.8
Sister	12	12.5
Other relatives	6	6.3
Friend	3	3.1
**EPDS**		
EPDS ≥ 13	33	16.5
EPDS < 13	167	83.5
**PSQI**		
PSQI > 5 Poor sleep quality	32	16.0
PSQI ≤ 5 Good sleep quality	168	84.0

*Note* EPDS: Edinburgh Postnatal Depression Scale, PSQI: Pittsburgh Sleep Quality Index

### Descriptive results of breastfeeding self-efficacy

When the difference between breastfeeding self-efficacy using descriptive characteristics of women in the postpartum phase was examined, data not given in the table, a statistically significant difference was found only between the number of births and BSES-SF scores (*F* = 3.68; *p* = 0.001). According to Bonferroni-corrected post hoc analysis results, BSES-SF scores of women who gave birth for the first time were found to be lower. A statistically significant difference was not found among age, educational- working status of both parents, staying with a relative after birth, planned pregnancy, mode of delivery, having problems during pregnancy or breastfeeding self-efficacy (*p* > 0 0.05).

### Breastfeeding self-efficacy, sleep quality, perceived social support and depression results

Table 2 shows the BSES-SF, PSQI, MSPSS and EPDS total and subscale mean results of the participants. The mean BSES-SF score of the participants was 59.05 ± 8.28; the mean PSQI total score was 9.18 ± 3.67; the mean MSPSS total score was 57.82 ± 18.81; and EPDS mean score was 8.98 ± 5.89 (Table 2).

**Table 2:** Descriptive statistics for BSES-SF, PSQI, MSPSS, and EPDS scores

Scale’s	Scale’s Min–Max scores	Participants’ Min–Max scores	Mean	SD
BSES**-**SF	14–70	34–70	59.05	8.28
PSQI total	0–21	1–21	9.18	3.67
Sleep quality	0–3	0–3	1.45	0.87
Sleep latency	0–3	0–3	1.15	0.99
Sleep duration	0–3	0–3	1.84	1.07
Sleep efficiency	0–3	0–3	2.28	1.03
Sleep disturbances	0–3	0–3	1.56	0.76
Use of sleep medication	0–3	0–3	0.11	0.46
Daytime dysfunction	0–3	0–3	0.79	0.92
MSPSS total	12–84	12–84	57.82	18.81
Family support	4–28	4–28	23.87	5.82
Friend support	4–28	4–28	17.34	8.45
Other person support	4–28	4–28	16.61	8.47
EPDS	0–30	0–26	8.98	5.89

*Note* BSES-SF: Breastfeeding Self-Efficacy Scale-Short Form, PSQI: Pittsburgh Sleep Quality Index, MSPSS: Multidimensional Scale of Perceived Social Support, EPDS: Edinburgh Postnatal Depression Scale

### Correlations among breastfeeding self-efficacy, sleep quality, perceived social support and depression

This study showed statistically significant negative correlations between PSQI total scores and BSES-SF scores (*r***= **-0.612, *p* = 0.004) and between EPDS and BSES-SF scores (*r***= **-0.445, *p* = 0.001) (*p* < 0.05). As the PSQI total and EPDS scores of women increase, BSES-SF scores decrease (Table 3).

**Table 3:** Correlation of BSES-SF with PSQI, MSPSS, and EPDS of the particapants

Scale’s	BSES-SF	
	r	p
PSQI total	-0.612	0.004*
MSPSS total	0.341	0.036*
Family support	0.373	0.014*
Friend support	0.065	0.361
Other person support	0.127	0.073
EPDS	-0.445	0.001*

*Note*. r: Pearson correlations test, BSES-SF: Breastfeeding Self-Efficacy Scale-Short Form, PSQI: Pittsburgh Sleep Quality Index, MSPSS: Multidimensional Scale of Perceived Social Support, EPDS: Edinburgh Postnatal Depression Scale, * *p* < 0.05

Statistically significant positive correlations were found between MSPSS total and BSES-SF scores (r **=** 0.341, *p* = 0.036) and between the family support subscale and BSES-SF scores (r **=** 0.373, *p* = 0.014) (*p* < 0.05). As the MSPSS total and family support subscale scores of women increase, their BSES-SF scores also increase (Table 3).

### Linear regression among self-efficacy, sleep quality, perceived social support, family support and depression

Multiple linear regression analysis was employed to ascertain whether sleep quality, perceived social support, family support, and depression significantly predict breastfeeding self-efficacy. Table 4 shows the linear regression results for the association among breastfeeding self-efficacy, sleep quality, perceived social support, family support and depression. According to the regression analysis the model was found to be significant (F = 58.867, *p* < 0.001, *R*^*2*^ = 0.548, adjusted *R*^*2*^ = 0.539). It was determined that 53.9% of the variance in breastfeeding self-efficacy (adjusted R^2^ = 0.539) is explained by sleep quality, perceived social support, family support, and depression. Results indicate that sleep quality (β=-0.491, *p* = 0.001), perceived social support (β = 0.146, *p* = 0.015), family support (β = 0.153, *p* = 0.013) and depression (β = -0.228, *p* = 0.001) significantly predict breastfeeding self-efficacy (*p* < 0.05) (Table 4).

**Table 4:** Multiple linear regression coefficients among breastfeeding self-efficacy, perceived social support, family support, and depression variables

Independent variables	B	SE_B_	95% CI for B		β	t	p
			Lower limit	Upper limit			
Constant	64.856	2.604	59.721	69.992	-	24.907	0.001*
**PSQI total**	-1.388	0.151	-1.686	-1.091	-0.491	-9.201	0.001*
**MSPSS total**	0.066	0.027	0.013	0.120	0.146	2.454	0.015*
Family support	0.234	0.094	0.050	0.419	0.153	2.505	0.013*
**EPDS**	-0.349	0.082	-0.511	-0.187	-0.228	-4.240	0.001*
Dependent variable: Breastfeeding self-efficacy							
R: 0.740 R^2^: 0.548 Adjusted R^2^:0.539 F: 58.867 p:0.001 Durbin-Watson: 2.096							

*Note* Beta: Unstandardised coefficients, β: Standardised coefficients, SEB: Standart error for B, CI: 95% Confidence Interval for B, BSES-SF: Breastfeeding Self-Efficacy Scale-Short Form, PSQI: Pittsburgh Sleep Quality Index, MSPSS: Multidimensional Scale of Perceived Social Support, EPDS: Edinburgh Postnatal Depression Scale, * *p* < 0.05

## Discussion

The current study was conducted to examine breastfeeding self-efficacy in postpartum women in terms of sleep quality, perceived social support, depression and other variables. This study revealed that women’s breastfeeding self-efficacy levels are high, and their thoughts and perceptions about breastfeeding are positive. They are self-confident and are willing to cope with problems that may occur in the breastfeeding process. Similarly, in studies conducted on postpartum women in Turkey and in different countries, breastfeeding self-efficacy levels of women were found to be above average [6–8, 21]. This study found that among the descriptive characteristics, only the number of births affected the results, revealing lower levels of breastfeeding self-efficacy among those giving birth for the first time. This is an expected result, as the number of births affects the breastfeeding experience and the woman’s self-confidence regarding breastfeeding. Previous research has addressed the relationship between breastfeeding self-efficacy and variables in the postpartum period, such as type of delivery, number of pregnancy/births, time to start breastfeeding, and social and demographic characteristics such as education, age and employment status. In some of these studies, descriptive features were found to be significant, and in others, no such effect was found [13, 21, 22]. In this study, it was found that only the number of births affected breastfeeding self-efficacy among the descriptive characteristics of women. This research revealed the need for counseling on breastfeeding for women who gave birth for the first time. This may be due to the fact that women giving birth for the first time need more counseling and support regarding the process.

According to their PSQI scores, the women’s sleep quality was moderate; 16% had poor sleep quality. This concurs with research conducted among postpartum women in Turkey, where sleep quality was also found to be moderate [9]. Various sleep quality rates have been reported in studies using the same measurement tool with postpartum women in different countries. For instance, in a study conducted with mothers in the third month postpartum period in Taiwan, 38.4% of mothers had poor sleep quality [14]. In China, the prevalence of poor maternal sleep quality was 48.8% in the third month and 49.2% in the sixth month [32]. In Nepal, for women 2–12 months postpartum, the prevalence of poor sleep quality was 28.2% [19]. Christian et al. (2019), in their study with African American and White women at 4–11 weeks postpartum, reported that 68.0–80.0% of women had poor overall sleep quality [33]. Another study focused on Hispanic and non-Hispanic primiparous mothers in the USA to determine six-month postpartum sleep quality and determined that the rate of poor sleep quality was 59.0% [34]. These different rates may be due to the characteristics of the sample group. However, in line with the results of studies, the literature indicates that women in the postpartum period are more likely to experience interrupted, fragmented and lower quality sleep due to having a newborn baby.

Current study has shown that sleep quality is one of the predictors of breastfeeding self-efficacy. According to this result, as the sleep quality of women increases, their breastfeeding self-efficacy also increases. Similarly, in research conducted with postpartum women in Turkey, a positive correlation was found between sleep quality and breastfeeding self-efficacy [9]. Chrzan-Dętkoś et al. (2021) stated that breastfeeding self-efficacy increased in postpartum women when their insufficient sleep problems decreased. In addition, studies show that breastfeeding is more successful in women with good sleep quality [19, 35]. This research shows the significance of sleep quality in the successful continuation of breastfeeding.

In current study we determined that the perceived social support of women was moderate, and the majority of support came from their families. This result can be explained by the strong family ties in Turkish culture. In this study perceived social support particularly family support were found predictors of breastfeeding self-efficacy. When the perceived social support and family support of women within the scope of our research increased, so did their breastfeeding self-efficacy. In studies conducted in Turkey using the same measurement tools, perceived social support and family support in postpartum women positively affected breastfeeding self-efficacy [7]. In China, it was found that family support and social support contributed greater to breastfeeding self-efficacy [36]. Ngo et al., (2019), in their study with postpartum Vietnamese women, determined that breastfeeding self-efficacy was positively correlated with perceived social support [8]. Social support was one indicator of breastfeeding self-efficacy in studies made in Iran with those who had babies under six months [18, 37]. Result of present research similarly shows that the social support perceived by women is a determining factor in continuation of breastfeeding.

This study determined that 16.5% of women had a high risk of postpartum depression. In studies conducted in Turkey using the EPDS measurement tool, the risk is between 13 and 51% [38, 39]. In Nepal, the prevalence of depression in women at 2–12 months postpartum period was 18.7% [19]; in Japan it was 7.6% at one-month postpartum [20]; and among American Indian/Alaska native women it was 14.0 – 29.7% [40]. In this study, another predictor of breastfeeding self-efficacy among postpartum women was depression. It was determined within the scope of this study that as the risk of postpartum depression increased in women, breastfeeding self-efficacy decreased. In a study conducted in Turkey, in a sample of women aged 15–49 years in their first 42 days of the postpartum period, it was determined that as the level of depression increases, the level of breastfeeding self-efficacy decreases [7]. Ngo et al., (2019) indicated that mothers with high levels of postpartum depression had lower breastfeeding self-efficacy [8]. Similarly, in this study, it was revealed that depression negatively affected breastfeeding self-efficacy, which shows the importance of the mother’s self-confidence and determination to breastfeed.

Nurses are key health professionals who are often in contact with women in the perinatal and postpartum periods. This is an important opportunity to examine postpartum women for breastfeeding self-efficacy and sleep quality, perceived social support and depression and to plan and implement preventative and treatment options. Therefore, in the perinatal period, before hospital discharge and during the postpartum period, nurses should evaluate postpartum women in terms of perceived social support, sleep quality, and depression and support breastfeeding. The fact that this study was conducted with women at 0–4 months postpartum, provides data on breastfeeding self-efficacy in this period. However, the cause and effect relationship could not be revealed completely because of the cross-sectional design of the study. In future studies, it will be important to conduct intervention and longitudinal studies to reveal the cause and effect relationship between breastfeeding self-efficacy of women in the postpartum period and the factors affecting them.

### Limitations

This study has some limitations. First, it was carried out in a tertiary hospital in Ankara, Turkey, and the sample is a purposive sample at one location, so it cannot be generalised beyond the sample. Second, the scores obtained from the scales used in the study are based on the statements of the women. The reliance on self-reported data may have affected the validity of the responses. Women between 0 and 4 months postpartum were included in this study, and this range may act as a confounder, thus affecting the results.

## Conclusions

This study found that postpartum women have a high understanding of their breastfeeding self-efficacy, a medium perception of sleep quality and social support, and a low risk of depression. The results of this study can enrich our knowledge of the role of sleep quality, perceived social support, and depression in influencing breastfeeding self-efficacy. In this study, sleep quality, perceived social support, and depression were the main predictors of breastfeeding self-efficacy. The increase in sleep quality and perceived social support positively affected the breastfeeding self-efficacy of postpartum women, while giving birth for the first time and an increase in the risk of depression were negatively affected. Self-efficacy is an effective factor in sustaining breastfeeding. Nurses can influence breastfeeding behaviour by strengthening the mother’s self-efficacy. In addition, women who gave birth for the first time had lower breastfeeding self-efficacy. Counseling on breastfeeding may be recommended for these women. Therefore, it is suggested that nurses evaluate postpartum women in terms of perceived social support, sleep quality, depression and number of births. Additionally, we believe that it would be useful to conduct longitudinal studies to evaluate breastfeeding self-efficacy in postpartum women.

## Data Availability

The datasets used and/or analysed during the current study is available from the corresponding author on reasonable request.

## Abbreviations

BSES-SF: Breastfeeding Self-Efficacy Scale-Short Form
PSQI: Pittsburgh Sleep Quality Index
MSPSS: Multidimensional Scale of Perceived Social Support
EPDS: Edinburgh Postnatal Depression Scale
WHO: World Health Organization
UNICEF: United Nations Children’s Fund
